# Supplementary material for: Tiered sympathetic control of cardiac function revealed by viral tracing and single cell transcriptome profiling
Source: eLife. 2023 May 10;12:e86295. doi: 10.7554/eLife.86295 (PMC10212561; doi:10.7554/eLife.86295)
Supplement: Supplementary file 1. [file elife-86295-supp1.docx]

### Supplementary Table 1

| Sample | Batch | Sequencing Depth  (UMIs/Cell) | Sequencing Saturation |
| --- | --- | --- | --- |
| Mouse-2-1_WTLM | 2 | 5,558 | 60.9% |
| Mouse-2-2_WTLM | 2 | 6,241 | 68.3% |
| Mouse-2-3_WTLM | 2 | 3,818 | 83.1% |
| Mouse-2-4_WTLM | 2 | 5,627 | 59.8% |
| Mouse-2-6_WTLM | 2 | 4,953 | 60.4% |
| Mouse-1-3_WTLM | 1 | 5,599 | 88.5% |
| Mouse-1-6_WTLM | 1 | 4,441 | 87.8% |
| Mouse-1-7_WTLM | 1 | 5,157 | 81.2% |
|  |  |  |  |
|  |  |  |  |
|  |  |  |  |
|  |  |  |  |
|  |  |  |  |
